# Data of Nebivolol on oxidative stress parameters in hypertensive patients

**DOI:** 10.1016/j.dib.2022.107913

**Published:** 2022-02-03

**Authors:** Helena C. Barroso, Murilo E. Graton, Simone R. Potje, Jéssica A. Troiano, Lilian X. Silva, Ana Cláudia M.S. Nakamune, Cristina Antoniali

**Affiliations:** a Multicenter Graduate Program of Physiological Sciences, SBFis, São Paulo State University (UNESP), Araçatuba, São Paulo, Brazil; b School of Dentistry, Department of Basic Sciences, São Paulo State University (UNESP), Araçatuba, São Paulo, Brazil; c Minas Gerais State University (UEMG), Passos, Minas Gerais, Brazil; d Faculdades de Dracena (UNIFADRA), Fundação Dracenense de Educação e Cultura, Curso de Medicina, Dracena, São Paulo, Brazil

**Keywords:** Nebivolol, Oxidative stress, Hypertension, Human beings

## Abstract

Oxidative stress is a key feature in hypertension, since reactive oxygen species are involved in all stages of cardiovascular diseases. Saliva is a body fluid that can be used to investigate alterations in the oxidative system with several specific advantages over blood. Nebivolol is a third-generation selective β1-adrenergic receptor antagonist that promotes vasodilation and has been shown to reduce oxidative stress in pre-clinical and clinical studies. The use of Nebivolol in different periods of treatment demonstrated that it is an efficient anti-hypertensive drug. We evaluated the oxidative stress biomarkers and the enzymatic and non-enzymatic antioxidant systems in saliva of hypertensive patients before and after the use of anti-hypertensive therapeutic doses of Nebivolol, since saliva can be used as an auxiliary tool to analyze parameters of oxidative stress.

## Specifications Table

**Table:** 

Subject	Cardiology and Cardiovascular Medicine
Specific subject area	Cardiovascular Pharmacology
Type of data	Table Graph Figure
How the data were acquired	Blood pressure measurements were collected using standard aneroid sphygmomanometer (WA5090-02, Welch Allyn Tycos®). Electrocardiographic data were recorded using ECGV6 software (ECG V6, HeartWare, Brazil). Waist circumference of every individual was collected using anthropometric metallic tape with a 0.1 cm scale and body mass index was taken on electronic balance (Welmy W200A). Biochemical analysis was performed through colorimetric tests.
Data format	Raw Analyzed
Description of data collection	Blood samples from twenty-four patients were collected in tubes containing ethylenediaminetetraacetic acid (EDTA). Saliva samples were collected before and at the end of Nebivolol treatment in humans at morning period (9–12 pm) using Salivette® (Genese Diagnosis, Germany), 2 h after fasting and oral hygiene with water and no toothbrush.
Data source location	• Institution: São Paulo State University (UNESP) • City: Araçatuba, São Paulo • Country: Brazil • Region: South America
Data accessibility	The data are presented in this article. Direct URL to Supplementary Material: http://hdl.handle.net/11449/216041

## Value of the Data

• The data reinforces the effect of the Nebivolol as antioxidant medication in the treatment of hypertension with significant reduction of systolic and diastolic blood pressure, also decreased heart rate and PR interval.
• The treatment of hypertension with drugs such as Nebivolol that improve the glycolipid profile, reduce oxidative stress, soften endothelial dysfunction, and which has good tolerability being cost-effective, is highly attractive for hypertensive patients. Furthermore, we observed that Nebivolol reduced serum creatinine levels, suggesting an improvement of kidney function, especially in patients with renal chronic failure.
• The data presented here could be the reference for future studies that plan the use of Nebivolol as a first choice for the treatment of cardiovascular diseases.

## 1 Data Description

Characteristics of hypertensive patients are described in Table 1. Male and female patients presented higher values of waist circumference than those recommended by The International Diabetes Federation [1]. After the treatment with Nebivolol, male patients reduced their values of waist circumference (Table 2). Ideal BMI was observed in three patients, and the other 21 remaining patients showed overweight and obesity of varying degrees. After treatment with Nebivolol, all patients decreased their BMI values (Table 3). Values of clinical, biochemical, and electrocardiographic parameters were synthesized in Table 4. The raw data regarding Tables 2, 3 and 4 are shown as Supplementary Material.

**Table 1:** Characteristics of studied groups including age, sex, race, body mass index, comorbidities, medication used concomitant, systolic blood pressure (SBP, mmHg) and diastolic blood pressure (DBP, mmHg) of the patients Untreated (before) and Treated (after) with Nebivolol.

Patient ID	Age (years)	Sex	Race	Body Mass Index (BMI, kg/cm^2^)	Comorbidities	Medication used 24 h before Nebivolol use	Changes on therapeutic scheme (A – added/ R – removed)	SBP X DBP (mmHg) from Untreated patients	SBP X DBP (mmHg) from Treated patients
**1**	58	Woman	White	41.7	Obesity	Losartan potassium (twice/day)	A: Nebivolol (5 mg/day)	160 × 100	140 × 90
**2**	40	Woman	White	22.1	Myocardial bypass for anterior descending artery; Dyslipidemia.	Oral contraceptive. Ezetimibe. Atenolol (25 mg/day).	A: Nebivolol (5 mg/day). R: Atenolol (25 mg/day).	140 × 85	120 × 80
**3**	46	Man	White	29.9	Dyslipidemia	None	A: Nebivolol (5 mg/day).	150 × 100	130 × 90
**4**	40	Woman	White	25.3	Dyslipidemia	None	A: Nebivolol (5 mg/day).	135 × 85	120 × 80
**5**	43	Woman	Yellow	32	Dyslipidemia	Hydrochloric-thiazide. Losartan potassium (50 mg/day).	A: Nebivolol (5 mg/day).	130 × 80	130 × 80
**6**	56	Woman	White	21.5	Mild depression; Menopause.	None	A: Nebivolol (5 mg/day).	140 × 95	120 × 80
**7**	44	Man	White	35.8	Obesity	None	A: Nebivolol (5 mg/day).	150 × 95	130 × 80
**8**	54	Man	White	37	Obesity; Dyslipidemia; Gout.	Allopurinol (300 mg). Ramipril 10/5 mg.	A: Nebivolol (5 mg/day).	150 × 100	120 × 80
**9**	37	Woman	White	26.1	None	Indapamide (1.5 mg/day). Nifedipine (30 mg/day).	A: Nebivolol (5 mg/day).	140 × 95	120 × 80
**10**	68	Man	White	35.3	Obesity; Dyslipidemia; Diabetes Mellitus II.	Hydrochloric-thiazide (12.5 mg/day). Losartan potassium (100 mg/day). Amlodipine (5 mg/day). Sinvastatina (20 mg/day) Metformin (1 g/day).	A: Nebivolol (5 mg/day).	140 × 100	130 × 80
**11**	44	Man	Black	31.6	None	Indapamide (1.5 mg).	A: Nebivolol (5 mg/day).	160 × 100	130 × 80
**12**	54	Woman	White	28.8	Dyslipidemia	Indapamide (1.5 mg).	A: Nebivolol (5 mg/day).	150 × 95	120 × 70
**13**	57	Woman	White	25.2	None	Losartan potassium (50 mg/day).	A: Nebivolol (5 mg/day). R: Losartan potassium (50 mg/day).	140 × 90	120 × 80
**14**	65	Woman	White	36	Obesity; Dyslipidemia; Moderate carotid artery stenosis.	Valsartan (320 mg/day). Hydrochloro-thiazide (12.5 mg/day). Amlodipine (5 mg/day). Rosuvastatin (10 mg/day). Acetylsalicylic acid (10 mg/day).	A: Nebivolol (5 mg/day).	160 × 100	130 × 90
**15**	63	Man	White	29.3	Dyslipidemia; Chronic kidney disease.	Amlodipine (5 mg/day). Hydrochloro-thiazide (12.5 mg/day). Losartan potassium (100 mg/day).	A: Nebivolol (5 mg/day).	150 × 100	120 × 80
**16**	43	Man	White	35.2	Obesity; Dyslipidemia; Mitral valve prolapse.	None	A: Nebivolol (5 mg/day).	140 × 100	130 × 80
**17**	62	Man	White	25.5	Dyslipidemia	Losartan potassium (100 mg/day).	A: Nebivolol (5 mg/day).	150 × 100	140 × 80
**18**	56	Woman	White	32.5	Obesity; Dyslipidemia; Diabetes Mellitus II; Mild depression; Chronic labyrinth-path.	Losartan potassium (100 mg/day). Amlodipine (5 mg/day). Hydrochloro-thiazide (12.5 mg/day). Paroxetin (20 mg/day). Linagliptin (5 mg/day). Metformin (1 g/day). Gingko Biloba (160 mg/day).	A: Nebivolol (5 mg/day).	140 × 95	120 × 80
**19**	25	Man	White	32.1	Obesity; Dyslipidemia.	None	A: Nebivolol (5 mg/day).	140 × 95	120 × 80
**20**	53	Man	White	28.7	None	Losartan potassium (100 mg/day). Hydrochloro-thiazide (12.5 mg/day).	A: Nebivolol (5 mg/day).	150 × 100	130 × 80
**21**	54	Man	White	27.9	Obesity; Dyslipidemia; Diabetes Mellitus II.	Losartan potassium (50 mg/day). Metformin (500 mg/day). Ciprofibrate (100 mg/day)	A: Nebivolol (5 mg/day).	140 × 90	120 × 80
**22**	33	Woman	White	34	Obesity; Dyslipidemia; Chronic lymphocytic thyroiditis.	Oral contraceptive. Sertraline (25 mg/day). Atenolol (25 mg/day). Levothyroxine (75 µg/ day). Indapamide (1.5 mg).	A: Nebivolol (5 mg/day). R: Atenolol (25 mg/day).	120 × 80	120 × 70
**23**	67	Woman	White	26.9	Dyslipidemia	Losartan potassium (100 mg/day). Olmesartan (40 mg/day). Rosuvastatin (5 mg/day). Propranolol (160 mg/ day).	A: Nebivolol (5 mg/day). R: Propranolol (160 mg/ day).	120 × 80	120 × 80
**24**	70	Man	Yellow	24.1	Dyslipidemia; Diabetes Mellitus II; Chronic peripheral vascular disease.	Metformin (500 mg/day). Rosuvastatin (20 mg/day). Acetylsalicylic acid and magnesium carbonate (81/24.30 mg/day). Spironolactone (25 mg/day).	A: Nebivolol (5 mg/day).	160 × 105	120 × 80

**Table 2:** Waist circumference, in centimeters, of the patients Untreated (before) and Treated (after) with Nebivolol. Data are represented as Mean ± SD and *n* represents the number of patients.

	Men		Women	
Sex	(*n* = 12, *p* = 0.0003)		(*n* = 12, *p* = 0.0050)	
Groups	Untreated	Treated	Untreated	Treated
Mean ± SEM	107.3 ± 10.18	103.8 ± 10.18*	94.1 ± 13.56	91.3 ± 13.69*

*p < 0.05.

**Table 3:** Body mass index (BMI), in kg/cm^2^, of the patients Untreated (before) and Treated (after) with Nebivolol. Data are represented as Mean ± SD and *n* represents the number of patients.

		Untreated Mean ± SD, *n*	Treated Mean ± SD, *n*	*p* value
BMI (Kg/cm^2^)	Total	30.15 ± 5.14, 24	29.17 ± 5.34, 24*	<0.0001
	≥ 25	31.24 ± 4.50, 21	30.22 ± 4.83, 21*	<0.0001
	< 25	22.57 ± 1.36, 3	21.80 ± 1.34, 3*	0.0009

*p < 0.05.

**Table 4:** Clinical, biochemical and electrocardiographic parameters in patients Untreated (before) and Treated (after) with Nebivolol. Data are represented as Mean ± SD and *n* represents the number of patients.

Variable		Untreated Mean ± SD, *n*	Treated Mean ± SD, *n*	*p* value
Systolic blood pressure (mmHg)		142.50 ± 12.94, 24	125.4 ± 5.88*, 24	<0.0001

Diastolic blood pressure (mmHg)		93.9 ± 7.65, 24	80.4 ± 3.58*, 24	<0.0001

Heart rate (bpm)	Total	76.38 ± 7.31, 24	67.00 ± 6.71*, 24	<0.0001
	≥ 80	84.00 ± 4.52, 9	72.22 ± 7.12*, 9	0.0008
	< 80	71.80 ± 4.05, 15	63.87 ± 4.13*, 15	<0.0001

Glucose (mg/dL)	Total	95.71 ± 19.28, 24	89.45 ± 10.01*, 24	0.0406
	≥ 100	141.00 ± 15.39, 3	100.0 ± 14.80*, 3	0.0035
	< 100	89.24 ± 7.21, 21	88.05 ± 8.64, 21	0.2993

Total cholesterol (mg/dL)	Total	193.30 ± 39.11, 24	181.30 ± 33.64*, 24	0.0032
	> 200	236.70 ± 21.51, 9	214.00 ± 18.19*, 9	0.0025
	≤ 200	167.30 ± 17.81, 15	161.60 ± 23.80, 15	0.1265

HDL (mg/dL)	Total	44.38 ± 2.3, 24	45.46 ± 2.4, 24	0.2232
	< 40	35.20 ± 3.04, 10	36.50 ± 4.60, 10	0.1717
	≥ 40	50.39 ± 10.98, 14	51.86 ± 11.54, 14	0.3437

LDL (mg/dL)	Total	116.10 ± 36.65, 24	102.10 ± 33.62*, 24	0.0011
	> 100	133.40 ± 31.65, 16	117.30 ± 30.60*, 16	0.0044
	≤ 100	81.50 ± 14.74, 8	71.75 ± 11.18, 8	0.0742

Triglycerides (mg/dL)	Total	156.70 ± 71.34, 24	152.20 ± 64.13, 24	0.3530
	> 150	221.90 ± 48.10, 11	193.50 ± 67.29, 11	0.1071
	≤ 150	101.50 ± 24.98, 13	117.20 ± 34.90, 13	0.0568

Creatinine (mg/dL)	Total	0.92 ± 0.28, 24	0.86 ± 0.22*, 24	0.0042
	≥ 1	1.24 ± 0.26, 8	1.10 ± 0.19*, 11	0.0073
	< 1	0.76 ± 0.11, 16	0.74 ± 0.09, 16	0.1092

Plasmatic Uric acid (mg/dL)	Total	6.15 ± 1.81, 24	5.58 ± 1.89*, 24	0.0043
	≥ 6	7.54 ± 1.11, 13	6.79 ± 1.61*, 13	0.0156
	< 6	4.42 ± 0.62, 9	4.08 ± 0.88, 9	0.1303

Interval PR (ms)	Total	155.00 ± 19.78, 24	165.80 ± 21.65*, 24	0.0045
	≥ 160	174.50 ± 9.34, 11	178.20 ± 22.72, 11	0.2942
	< 160	138.50 ± 5.54, 13	155.40 ± 14.50*, 13	0.0004

Interval QRS (ms)	Total	92.50 ± 9.89, 24	97.08 ± 14.29*, 24	0.0024
	≥100	103.30 ± 5.00, 9	111.10 ± 12.69*, 9	0.0116
	<100	86.00 ± 5.07, 15	88.67 ± 6.39, 15	0.0519

Interval QT (ms)	Total	384.60 ± 16.15, 24	395.00 ± 17.19*, 24	0.0109
	≥ 380	391.10 ± 11.00, 19	395.80 ± 16.10, 19	0.1128
	< 380	360.00 ± 0.00, 5	392.0 ± 22.80*, 5	0.0175

*p < 0.05.

We observed a significant reduction in SBP and DBP before and after the treatment ensuring the antihypertensive effects of Nebivolol. There was significant reduction of heart rate after use of the Nebivolol.

The clinical analysis of biochemical parameters showed significant reduction in serum glucose, total cholesterol, LDL, creatinine, and uric acid after eight weeks of treatment with Nebivolol. These biochemical parameters alterations were more expressive in patients exhibiting, before treatment with Nebivolol, values of glucose, total cholesterol, LDL, creatinine, and uric acid greater than or equal to 100, 200, 100, 1.0, and 6.0 mg/dL, respectively. However, triglycerides and HDL levels were not significantly altered by treatment with Nebivolol.

Electrocardiograms demonstrated an increase in PR interval, duration of the QRS complex, and QT interval, but these values continued the boards of normality with the established treatment.

**Fig. 1:**
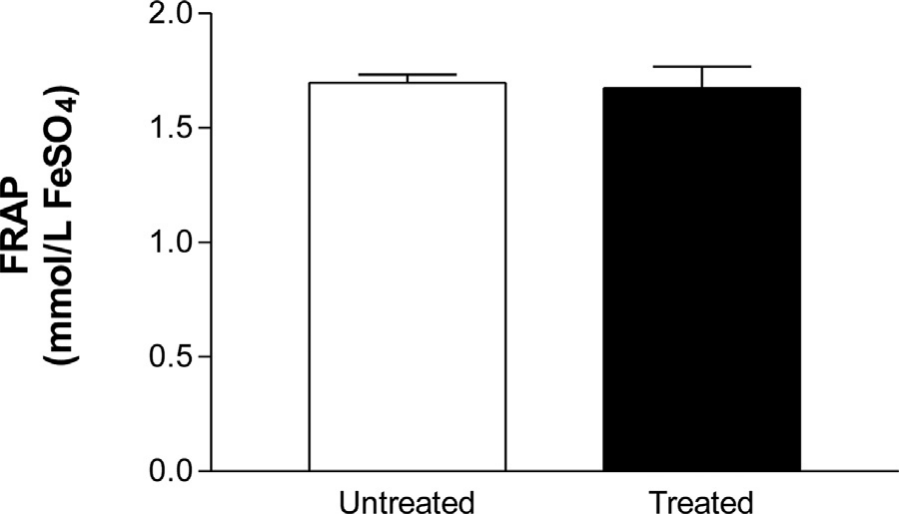
Ferric reducing antioxidant power (FRAP) assay, in mmol/L FeSO_4,_ in saliva of patients before (untreated, white bar) and after (treated, black bar) treatment with Nebivolol (5 mg/ day/ 8 weeks). Bars represent the Mean ± SD of the values.

**Fig. 2:**
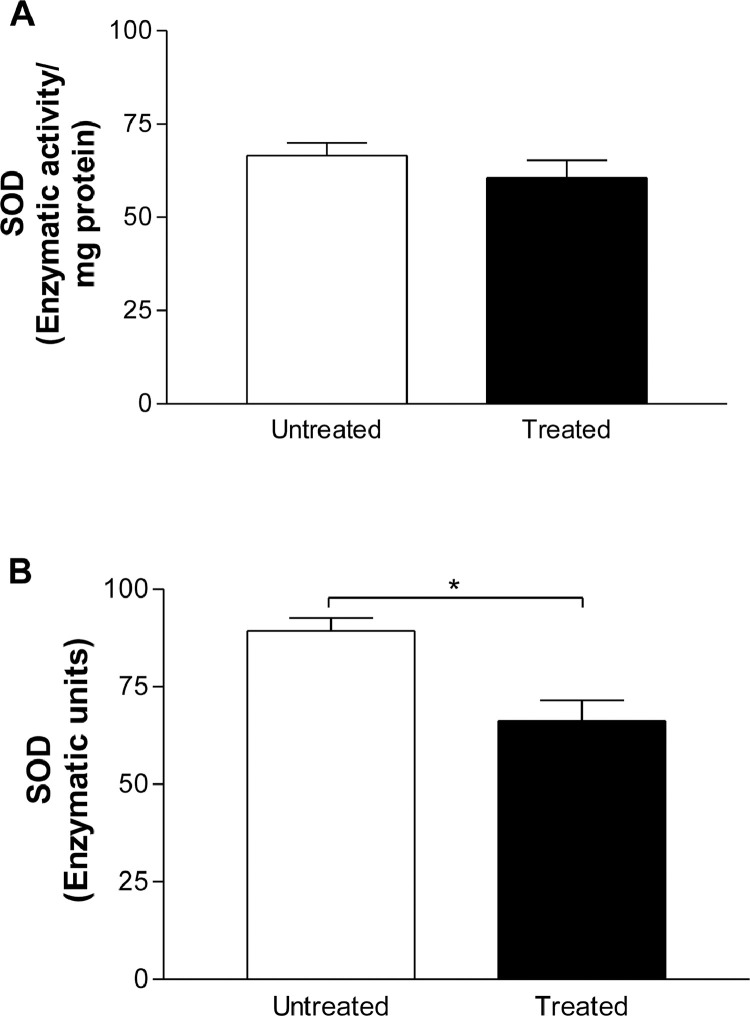
Superoxide dismutase (SOD) activity in (A) enzymatic activity/ mg protein, and (B) enzymatic units in saliva of patients before (untreated, white bar) and after (treated, black bar) treatment with Nebivolol (5 mg/ day/ 8 weeks). Bars represent the Mean ± SD of the values. *p < 0.05 SOD Enzymatic Units – Treated *versus* Untreated.

**Fig. 3:**
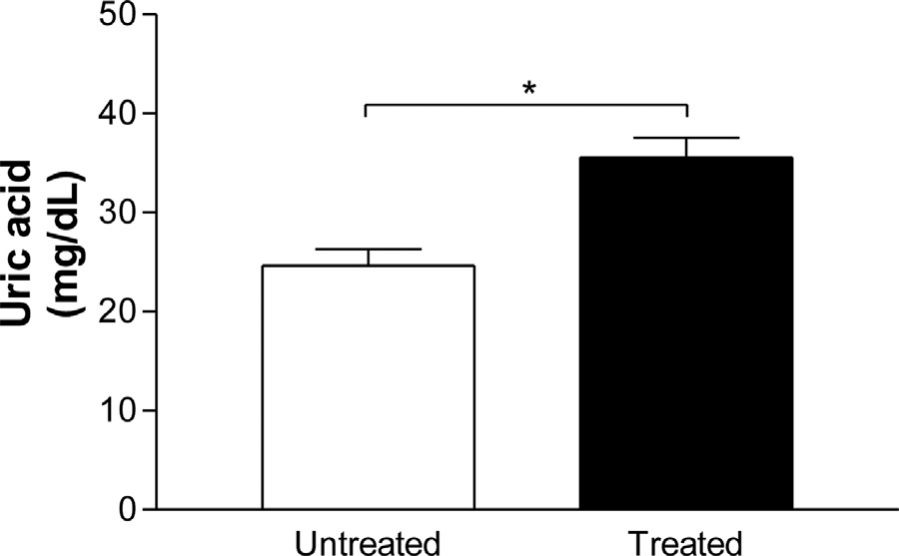
Uric acid, in mg/mL, in saliva of patients before (untreated, white bar) and after (treated, black bar) treatment with Nebivolol (5 mg/ day/ 8 weeks). Bars represent the Mean ± SD of the values. *p < 0.05 Treated *versus* Untreated.

**Fig. 4:**
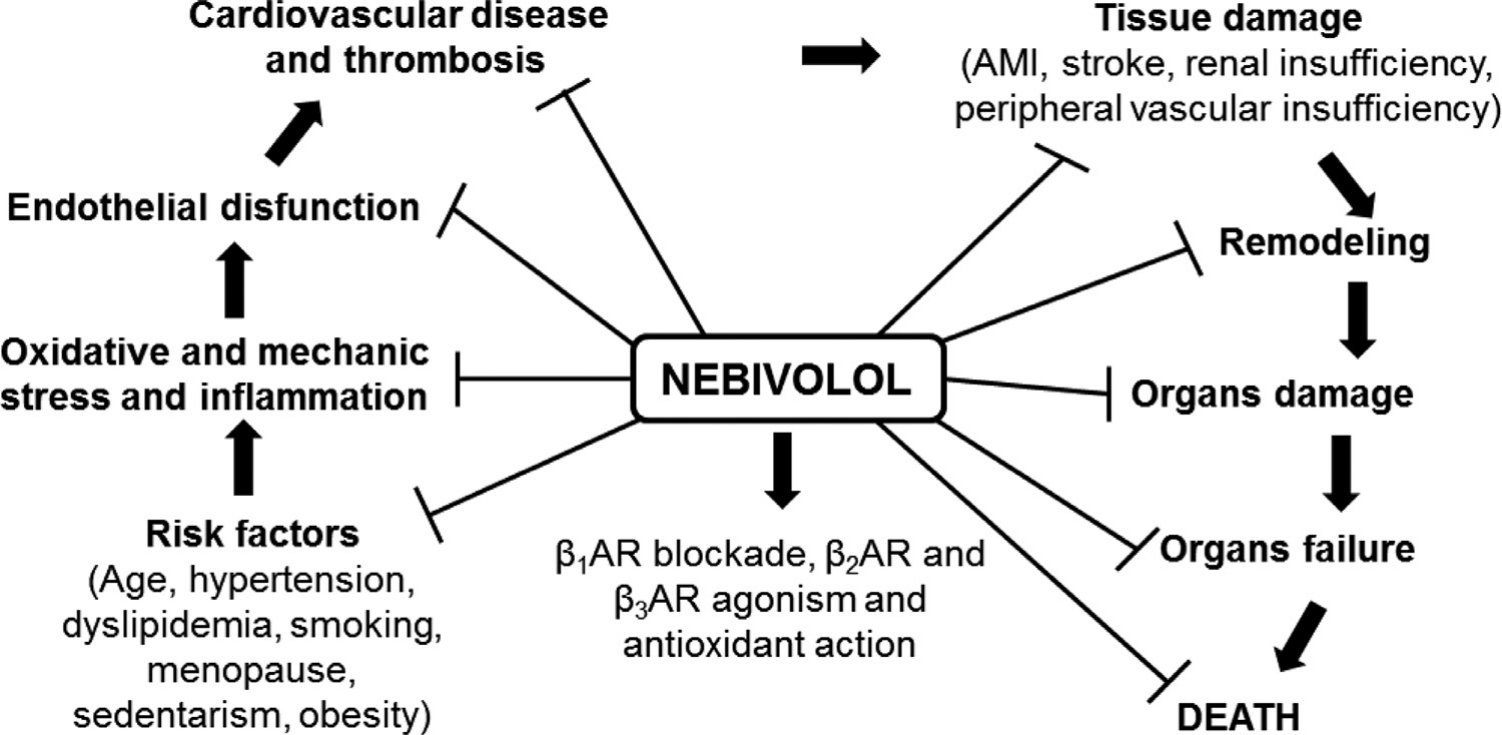
Influence of Nebivolol on the cardiovascular disease *continuum*.

The total antioxidant capacity did not alter in saliva of the patients before (1.697 ± 0.4334, *n* = 24) and after (1.674 ± 0.09285 mmol/L, *n* = 24) treatment with Nebivolol (Fig. 1). The salivary enzymatic units of SOD were slightly decreased by Nebivolol (untreated: 89.32 ± 4.87, *n* = 7; treated: 66.17 ± 7.87, *n* = 7) (Fig. 2B), however when we normalized these results, we did not observe difference on the salivary SOD activity before (66.55 ± 4.97, *n* = 7) and after (60.66 ± 6.37, *n* = 8) Nebivolol treatment (Fig. 2A). On the other hand, the UA values were raised in saliva of patients after treatment with Nebivolol (untreated: 24.6 ± 1.686, *n* = 18; treated: 35.56 ± 2; 014, *n* = 22, mg/mL) (Fig. 3). Graphical abstract of Nebivolol's influence on the cardiovascular disease *continuum* can be seen in Fig. 4.

## 2 Experimental Design, Materials and Methods

### 2.1 Study populations

The study is a prospective cohort study involving 24 patients (12 women and 12 men) aged 25–70 years (51.0 ± 2.3 years, *n* = 24) from Araçatuba (São Paulo, Brazil) where the sample group was determined by convenience. Patients were selected presenting stage 1 or 2 hypertension (showing at baseline, systolic blood pressure (SBP) < 180 mmHg and diastolic blood pressure (DBP) < 110 mmHg) [2]. These patients were using or not other antihypertensive agents, with or without medications for another associated comorbidities (diabetes mellitus, dyslipidemia, obesity, minor depression, gout, myocardial bridge, chronic peripheral vascular insufficiency and coronary artery disease). Previously prescribed drugs were kept without additional drugs. The 30 min of moderate-intensity aerobic activity, 3 times a week, was also maintained. The patients presented good oral hygiene, no periodontal diseases or gingival inflammation and the usual diet was kept (low salt and there was no significant change in dietary pattern during the study). In addition, patients self-reported their ethnic according to the classification of the Ethnic-Racial Characteristics of the Brazilian Population guidelines [3], organized by the Brazilian Institute of Geography and Statistics, where Brazilian populations are classified in races as: White, Black, Brown, Yellow and Indigenous. More details about the studied population can be seen on Table 1.

Patients were identified before (untreated) and after treatment with Nebivolol (treated). These patients received numeric ID in chart prepared for follow-up during the project development, containing name, sex, body weight (kg), height (meters), the body mass index (BMI*,* Kg/m^2^ of body surface area), the measurement of waist circumference (WC, cm), blood pressure (BP, mmHg), electrocardiographic data (ms and bpm), the biochemistry of blood (mg/dL). Moreover, other prerequisites were included during the execution of the project as drugs in previous use, dose, and associated comorbidities.

All individuals were evaluated by full medical history and clinical examination with laboratory investigations to exclude any other systemic or local disease that may affect the parameters examined in this study. Patients using drugs or supplements (nonsteroidal anti-inflammatory, immunosuppressive, corticosteroid or vitamin A, C and E therapy), smokers, excessive alcohol consumers, postmenopausal woman with hormone replacement therapy, hypertensive patients with BP above 180/110 mmHg, atrial fibrillation or cardiac pacemaker (artificial heart command), as well as those who have classical contraindications to the use of β-blockers or oral cavity diseases (excluded by direct inspection) were excluded of the study. The cardiologist HCB made all the evaluations (CRM: 63505).

### 2.2 Treatment

Patients received 5 mg orally daily of Nebivolol (Nebivolol hydrochloride, Nebilet®, Pharmaceutical Biolab-Sanus, Brazil) for eight weeks. Nebivolol has been added to a prior drug scheme to achieve therapeutic goal (SBP < 140 and DBP < 90 mmHg), as a replacement of a previous antihypertensive drug (in case of adverse effects where the Nebivolol would have better tolerability) or as initial antihypertensive therapy. All parameters were evaluated before and after treatment with Nebivolol in the same patient.

### 2.3 Clinical parameters: blood pressure evaluation

SBP and DBP were measured with an auscultatory method by using a standard sphygmomanometer (Tycos, USA, previously validated and calibrated by INMETRO-Instituto Nacional de Metrologia, Qualidade e Tecnologia) before and after treatment with Nebivolol.

Three measures were made in both arms, with 1-minute interval, in a sitting position, after 3–5 min of rest, using the average of the results obtained in each patient [2].

### 2.4 Electrocardiographic (ECG) assessment

Measurements were made after 15 min of rest. The standard twelve-lead ECGs were recorded with a paper speed of 25 mm/s and the voltage was 1 mm/mV, using ECGV6 software (ECG V6, HeartWare, Brazil). ECG wave's analysis were done digitally and conferred manually to calculate heart rate (HR, bpm), QRS and PR/QT interval duration (ms). The R-R interval was measured and used to compute the heart rate and to correct the QT interval (QTc) with Bazett's formula: QT_c_ = QT/√RR interval [4].

### 2.5 Biochemical analysis

Two blood samples were taken after a period of 12 h fasting to evaluate plasma glucose, total cholesterol, high-density lipoprotein cholesterol (HDL), low-density lipoprotein cholesterol (LDL), triglycerides, uric acid, and creatinine in all patients by standard clinical laboratory methods according to certified assays, in a clinical investigation laboratory of Araçatuba.

### 2.6 Saliva collection

Unstimulated saliva was collected at morning period (9–12 pm) during 5 min using Salivette® (Genese Diagnosis, Germany), 2 h after fasting and oral hygiene with water and no toothbrush. Salivary samples were kept on ice and then centrifuged at 5500 × g for 10 min (Centrifuge 5810R, rotor S-4-104, Eppendorf, Germany), as previously described [5]. Supernatants were fractionated and stored at −80°C until the experiments. Saliva samples were collected from each patient before and after treatment with Nebivolol.

### 2.7 Salivary total antioxidant capacity

The ferric reducing antioxidant power (FRAP) assay was used to assess salivary total antioxidant capacity. This method is based on reducing the ferric complex tripyridyl triazine (Fe^3+^TPTZ) to form Fe^2+^ in acidic medium [6]. An aliquot of saliva (15 µL) was used, and the absorbance was determined at 595 nm (Hitachi U1100 spectrophotometer, Japan), using a standard curve of ferrous sulfate (FeSO_4_). The results were expressed in µmol/L FeSO_4_.

### 2.8 Superoxide dismutase (SOD) activity

An aliquot of saliva (20 µL) previously diluted in Tris's solution (1:10 v/v) was used to determinate SOD activity based on the inhibition of the pyrogallol autoxidation as previously described [7]. The amount of enzyme required to inhibit 50% of the autoxidation of pyrogallol was considered as a unit of enzyme activity. Results were expressed in mg/protein.

### 2.9 Uric acid (UA)

UA levels in saliva were determined using a commercial kit (Labtest Diagnóstica SA, Brazil) based on the colorimetric enzymatic Trinder method, following the manufacturer's instructions, at 546 nm (Hitachi U1100 spectrophotometer, Japan). The results were expressed in mg/dL.

### 2.10 Statistical analysis

Data were expressed as mean ± standard deviation (SD). We performed the normality of the data using the Shapiro-Wilk test. Statistical analysis of the results was performed using paired Student's *t*-test (GraphPad Prism, 3.0, USA). Differences were significant when *p* < 0.05.

## Ethics Statement

This study was approved by the Human Ethics Committee of School of Dentistry, Araçatuba, São Paulo State University - UNESP (CAAE 53192116.4.0000.5420), and it was performed in compliance with the recommendations of the Declaration of Helsinki. All participants signed a free and informed consent form prior to participation.

## CRediT authorship contribution statement

**Helena C. Barroso:** Conceptualization, Methodology, Data curation, Writing – original draft. **Murilo E. Graton:** Methodology, Formal analysis, Writing – original draft. **Simone R. Potje:** Formal analysis, Writing – original draft, Writing – review & editing. **Jéssica A. Troiano:** Methodology, Writing – original draft, Writing – review & editing. **Lilian X. Silva:** Methodology, Formal analysis. **Ana Cláudia M.S. Nakamune:** Methodology, Resources, Writing – original draft. **Cristina Antoniali:** Conceptualization, Resources, Supervision, Writing – original draft, Writing – review & editing, Funding acquisition.

## Declaration of Competing Interest

The authors declare that there are no conflicts of interest.

## Appendix Supplementary Materials

Image, application 1

Image, application 2

Image, application 3
