# Supplementary material for: Data of Nebivolol on oxidative stress parameters in hypertensive patients
Source: Data Brief. 2022 Feb 3;41:107913. doi: 10.1016/j.dib.2022.107913 (PMC8847804; doi:10.1016/j.dib.2022.107913)
Supplement: Supplementary file 1 [file mmc1.doc]

**Supplementary Material**

| **Supplementary Table 1 – Waist circumference, in centimeters, of the patients Untreated (before) and Treated (after) with Nebivolol. Raw data collected.** | | | | |
| --- | --- | --- | --- | --- |
| Sex | Men | | Women | |
| Group | Untreated | Treated | Untreated | Treated |
| Waist circumference (cm) | 115.0 | 120.0 | 102.0 | 118.0 |
|  | 118.0 | 83.0 | 101.0 | 81.0 |
|  | 114.0 | 98.0 | 96.0 | 95.0 |
|  | 118.0 | 96.0 | 113.0 | 95.0 |
|  | 109.0 | 93.0 | 98.0 | 86.0 |
|  | 101.0 | 82.0 | 116.0 | 83.0 |
|  | 117.0 | 115.0 | 103.0 | 113.0 |
|  | 101.0 | 102.0 | 114.0 | 93.0 |
|  | 104.0 | 97.0 | 113.0 | 99.0 |
|  | 111.0 | 88.0 | 114.0 | 82.0 |
|  | 88.0 | 78.0 | 86.0 | 75.0 |
|  | 92.0 | 78.0 | 90.0 | 76.0 |
