# Supplementary material for: Data of Nebivolol on oxidative stress parameters in hypertensive patients
Source: Data Brief. 2022 Feb 3;41:107913. doi: 10.1016/j.dib.2022.107913 (PMC8847804; doi:10.1016/j.dib.2022.107913)
Supplement: Supplementary file 2 [file mmc2.doc]

**Supplementary Material**

| **Supplementary Table 2 – Clinical, biochemical, and electrocardiographic parameters in patients Untreated (before) and Treated (after) with Nebivolol. Raw data collected.** | | | |
| --- | --- | --- | --- |
| Variable | | Untreated | Treated |
| Systolic blood pressure (mmHg) | | 160.0 | 130.0 |
|  |  | 140.0 | 130.0 |
|  |  | 150.0 | 130.0 |
|  |  | 130.0 | 120.0 |
|  |  | 120.0 | 120.0 |
|  |  | 140.0 | 120.0 |
|  |  | 150.0 | 130.0 |
|  |  | 150.0 | 130.0 |
|  |  | 120.0 | 120.0 |
|  |  | 140.0 | 130.0 |
|  |  | 160.0 | 130.0 |
|  |  | 150.0 | 120.0 |
|  |  | 140.0 | 120.0 |
|  |  | 160.0 | 130.0 |
|  |  | 150.0 | 120.0 |
|  |  | 140.0 | 130.0 |
|  |  | 150.0 | 140.0 |
|  |  | 140.0 | 130.0 |
|  |  | 140.0 | 120.0 |
|  |  | 150.0 | 130.0 |
|  |  | 140.0 | 120.0 |
|  |  | 120.0 | 120.0 |
|  |  | 120.0 | 120.0 |
|  |  | 160.0 | 120.0 |
| Diastolic blood pressure (mmHg) | | 100.0 | 80.0 |
|  |  | 80.0 | 80.0 |
|  |  | 100.0 | 90.0 |
|  |  | 85.0 | 80.0 |
|  |  | 80.0 | 80.0 |
|  |  | 95.0 | 80.0 |
|  |  | 100.0 | 80.0 |
|  |  | 100.0 | 80.0 |
|  |  | 90.0 | 80.0 |
|  |  | 100.0 | 80.0 |
|  |  | 100.0 | 80.0 |
|  |  | 95.0 | 80.0 |
|  |  | 90.0 | 80.0 |
|  |  | 100.0 | 90.0 |
|  |  | 100.0 | 80.0 |
|  |  | 100.0 | 80.0 |
|  |  | 100.0 | 80.0 |
|  |  | 95.0 | 80.0 |
|  |  | 95.0 | 80.0 |
|  |  | 100.0 | 80.0 |
|  |  | 90.0 | 80.0 |
|  |  | 80.0 | 70.0 |
|  |  | 80.0 | 80.0 |
|  |  | 100.0 | 80.0 |
| Heart rate  (bpm) | Total | 93.0 | 75.0 |
|  |  | 75.0 | 60.0 |
|  |  | 85.0 | 75.0 |
|  |  | 80.0 | 75.0 |
|  |  | 75.0 | 65.0 |
|  |  | 80.0 | 75.0 |
|  |  | 72.0 | 60.0 |
|  |  | 70.0 | 65.0 |
|  |  | 70.0 | 65.0 |
|  |  | 70.0 | 65.0 |
|  |  | 85.0 | 60.0 |
|  |  | 75.0 | 60.0 |
|  |  | 70.0 | 55.0 |
|  |  | 80.0 | 70.0 |
|  |  | 80.0 | 70.0 |
|  |  | 88.0 | 85.0 |
|  |  | 75.0 | 65.0 |
|  |  | 60.0 | 68.0 |
|  |  | 70.0 | 60.0 |
|  |  | 75.0 | 70.0 |
|  |  | 75.0 | 65.0 |
|  |  | 75.0 | 70.0 |
|  |  | 70.0 | 65.0 |
|  |  | 85.0 | 65.0 |
| Heart rate  (bpm) | ≥ 80 | 93.0 | 75.0 |
|  |  | 85.0 | 75.0 |
|  |  | 80.0 | 75.0 |
|  |  | 80.0 | 75.0 |
|  |  | 85.0 | 60.0 |
|  |  | 80.0 | 70.0 |
|  |  | 80.0 | 70.0 |
|  |  | 88.0 | 85.0 |
|  |  | 85.0 | 65.0 |
|  | < 80 | 75 | 60 |
|  |  | 75 | 65 |
|  |  | 72 | 60 |
|  |  | 70 | 65 |
|  |  | 70 | 65 |
|  |  | 70 | 65 |
|  |  | 75 | 60 |
|  |  | 70 | 55 |
|  |  | 75 | 65 |
|  |  | 60 | 68 |
|  |  | 70 | 60 |
|  |  | 75 | 70 |
|  |  | 75 | 65 |
|  |  | 75 | 70 |
|  |  | 70 | 65 |
| Glucose  (mg/dL) | Total | 93.0 | 90.0 |
|  |  | 84.0 | 83.0 |
|  |  | 74.0 | 97.0 |
|  |  | 87.0 | 81.0 |
|  |  | 96.0 | 83.0 |
|  |  | 94.0 | 80.0 |
|  |  | 86.0 | 78.0 |
|  |  | 86.0 | 94.0 |
|  |  | 81.0 | 88.0 |
|  |  | 145.0 | 110.0 |
|  |  | 98.0 | 86.0 |
|  |  | 89.0 | 96.0 |
|  |  | 77.0 | 83.0 |
|  |  | 124.0 | 83.0 |
|  |  | 98.0 | 78.0 |
|  |  | 98.0 | 94.0 |
|  |  | 87.0 | 92.0 |
|  |  | 154.0 | 107.0 |
|  |  | 86.0 | 75.0 |
|  |  | 96.0 | 91.0 |
|  |  | 99.0 | 111.0 |
|  |  | 85.0 | 88.0 |
|  |  | 85.0 | 82.0 |
|  |  | 95.0 | 99.0 |
|  |  |  |  |
| Glucose  (mg/dL) | ≥ 100 | 145.0 | 110.0 |
|  |  | 124.0 | 83.0 |
|  |  | 154.0 | 107.0 |
|  | < 100 | 93.0 | 90.0 |
|  |  | 84.0 | 83.0 |
|  |  | 74.0 | 97.0 |
|  |  | 87.0 | 81.0 |
|  |  | 96.0 | 83.0 |
|  |  | 94.0 | 80.0 |
|  |  | 86.0 | 78.0 |
|  |  | 86.0 | 94.0 |
|  |  | 81.0 | 88.0 |
|  |  | 98.0 | 86.0 |
|  |  | 89.0 | 96.0 |
|  |  | 77.0 | 83.0 |
|  |  | 98.0 | 78.0 |
|  |  | 98.0 | 94.0 |
|  |  | 87.0 | 92.0 |
|  |  | 86.0 | 75.0 |
|  |  | 96.0 | 91.0 |
|  |  | 99.0 | 111.0 |
|  |  | 85.0 | 88.0 |
|  |  | 85.0 | 82.0 |
|  |  | 95.0 | 99.0 |
| Total cholesterol  (mg/dL) | Total | 150.0 | 142.0 |
|  |  | 219.0 | 211.0 |
|  |  | 226.0 | 184.0 |
|  |  | 287.0 | 232.0 |
|  |  | 230.0 | 220.0 |
|  |  | 218.0 | 191.0 |
|  |  | 179.0 | 170.0 |
|  |  | 186.0 | 189.0 |
|  |  | 182.0 | 184.0 |
|  |  | 152.0 | 147.0 |
|  |  | 150.0 | 146.0 |
|  |  | 239.0 | 213.0 |
|  |  | 160.0 | 133.0 |
|  |  | 246.0 | 243.0 |
|  |  | 243.0 | 216.0 |
|  |  | 195.0 | 211.0 |
|  |  | 222.0 | 216.0 |
|  |  | 179.0 | 172.0 |
|  |  | 155.0 | 158.0 |
|  |  | 142.0 | 133.0 |
|  |  | 151.0 | 187.0 |
|  |  | 156.0 | 140.0 |
|  |  | 183.0 | 170.0 |
|  |  | 190.0 | 142.0 |
| Total cholesterol  (mg/dL) | > 200 | 219.0 | 211.0 |
|  |  | 226.0 | 184.0 |
|  |  | 287.0 | 232.0 |
|  |  | 230.0 | 220.0 |
|  |  | 218.0 | 191.0 |
|  |  | 239.0 | 213.0 |
|  |  | 246.0 | 243.0 |
|  |  | 243.0 | 216.0 |
|  |  | 222.0 | 216.0 |
|  | ≤ 200 | 150.0 | 142.0 |
|  |  | 179.0 | 170.0 |
|  |  | 186.0 | 189.0 |
|  |  | 182.0 | 184.0 |
|  |  | 152.0 | 147.0 |
|  |  | 150.0 | 146.0 |
|  |  | 160.0 | 133.0 |
|  |  | 195.0 | 211.0 |
|  |  | 179.0 | 172.0 |
|  |  | 155.0 | 158.0 |
|  |  | 142.0 | 133.0 |
|  |  | 151.0 | 187.0 |
|  |  | 156.0 | 140.0 |
|  |  | 183.0 | 170.0 |
|  |  | 190.0 | 142.0 |
| HDL  (mg/dL) | Total | 32.0 | 35.0 |
|  |  | 69.0 | 66.0 |
|  |  | 37.0 | 31.0 |
|  |  | 41.0 | 42.0 |
|  |  | 69.0 | 83.0 |
|  |  | 62.0 | 47.0 |
|  |  | 29.0 | 32.0 |
|  |  | 33.0 | 33.0 |
|  |  | 45.0 | 56.0 |
|  |  | 43.0 | 42.0 |
|  |  | 43.0 | 42.0 |
|  |  | 67.0 | 58.0 |
|  |  | 51.0 | 48.0 |
|  |  | 35.0 | 39.0 |
|  |  | 38.0 | 35.0 |
|  |  | 38.0 | 37.0 |
|  |  | 47.0 | 44.0 |
|  |  | 42.0 | 47.0 |
|  |  | 35.0 | 37.0 |
|  |  | 37.0 | 39.0 |
|  |  | 51.0 | 50.0 |
|  |  | 38.0 | 47.0 |
|  |  | 42.0 | 44.0 |
|  |  | 41.0 | 57.0 |
| HDL  (mg/dL) | < 40 | 32.0 | 35.0 |
|  |  | 37.0 | 31.0 |
|  |  | 29.0 | 32.0 |
|  |  | 33.0 | 33.0 |
|  |  | 35.0 | 39.0 |
|  |  | 38.0 | 35.0 |
|  |  | 38.0 | 37.0 |
|  |  | 35.0 | 37.0 |
|  |  | 37.0 | 39.0 |
|  |  | 38.0 | 47.0 |
|  | ≥ 40 | 69.0 | 66.0 |
|  |  | 41.0 | 42.0 |
|  |  | 69.0 | 83.0 |
|  |  | 62.0 | 47.0 |
|  |  | 45.0 | 56.0 |
|  |  | 43.0 | 42.0 |
|  |  | 43.0 | 42.0 |
|  |  | 67.0 | 58.0 |
|  |  | 51.0 | 48.0 |
|  |  | 47.0 | 44.0 |
|  |  | 42.0 | 47.0 |
|  |  | 51.0 | 50.0 |
|  |  | 42.0 | 44.0 |
|  |  | 41.0 | 57.0 |
| LDL  (mg/dL) | Total | 98.0 | 86.0 |
|  |  | 102.0 | 108.0 |
|  |  | 165.0 | 134.0 |
|  |  | 219.0 | 163.0 |
|  |  | 115.0 | 69.0 |
|  |  | 139.0 | 113.0 |
|  |  | 89.0 | 86.0 |
|  |  | 118.0 | 118.0 |
|  |  | 123.0 | 109.0 |
|  |  | 87.0 | 73.0 |
|  |  | 86.0 | 73.0 |
|  |  | 156.0 | 137.0 |
|  |  | 78.0 | 59.0 |
|  |  | 168.0 | 152.0 |
|  |  | 147.0 | 149.0 |
|  |  | 128.0 | 147.0 |
|  |  | 131.0 | 134.0 |
|  |  | 107.0 | 92.0 |
|  |  | 106.0 | 101.0 |
|  |  | 84.0 | 60.0 |
|  |  | 48.0 | 77.0 |
|  |  | 82.0 | 60.0 |
|  |  | 107.0 | 98.0 |
|  |  | 103.0 | 53.0 |
| LDL  (mg/dL) | > 100 | 102.0 | 108.0 |
|  |  | 165.0 | 134.0 |
|  |  | 219.0 | 163.0 |
|  |  | 115.0 | 69.0 |
|  |  | 139.0 | 113.0 |
|  |  | 118.0 | 118.0 |
|  |  | 123.0 | 109.0 |
|  |  | 156.0 | 137.0 |
|  |  | 168.0 | 152.0 |
|  |  | 147.0 | 149.0 |
|  |  | 128.0 | 147.0 |
|  |  | 131.0 | 134.0 |
|  |  | 107.0 | 92.0 |
|  |  | 106.0 | 101.0 |
|  |  | 107.0 | 98.0 |
|  |  | 103.0 | 53.0 |
|  | ≤ 100 | 89.0 | 86.0 |
|  |  | 87.0 | 73.0 |
|  |  | 86.0 | 73.0 |
|  |  | 78.0 | 59.0 |
|  |  | 84.0 | 60.0 |
|  |  | 48.0 | 77.0 |
|  |  | 82.0 | 60.0 |
|  |  | 89.0 | 86.0 |
| Triglycerides  (mg/dL) | Total | 97.0 | 101.0 |
|  |  | 240.0 | 189.0 |
|  |  | 123.0 | 97.0 |
|  |  | 109.0 | 136.0 |
|  |  | 234.0 | 342.0 |
|  |  | 88.0 | 157.0 |
|  |  | 83.0 | 78.0 |
|  |  | 177.0 | 194.0 |
|  |  | 74.0 | 98.0 |
|  |  | 87.0 | 73.0 |
|  |  | 110.0 | 162.0 |
|  |  | 81.0 | 145.0 |
|  |  | 155.0 | 133.0 |
|  |  | 290.0 | 162.0 |
|  |  | 290.0 | 156.0 |
|  |  | 145.0 | 135.0 |
|  |  | 221.0 | 193.0 |
|  |  | 147.0 | 169.0 |
|  |  | 70.0 | 102.0 |
|  |  | 105.0 | 71.0 |
|  |  | 260.0 | 302.0 |
|  |  | 180.0 | 167.0 |
|  |  | 162.0 | 140.0 |
|  |  | 232.0 | 151.0 |
| Triglycerides  (mg/dL) | > 150 | 240.0 | 189.0 |
|  |  | 234.0 | 342.0 |
|  |  | 177.0 | 194.0 |
|  |  | 155.0 | 133.0 |
|  |  | 290.0 | 162.0 |
|  |  | 290.0 | 156.0 |
|  |  | 221.0 | 193.0 |
|  |  | 260.0 | 302.0 |
|  |  | 180.0 | 167.0 |
|  |  | 162.0 | 140.0 |
|  |  | 232.0 | 151.0 |
|  | ≤ 150 | 97.0 | 101.0 |
|  |  | 123.0 | 97.0 |
|  |  | 109.0 | 136.0 |
|  |  | 88.0 | 157.0 |
|  |  | 83.0 | 78.0 |
|  |  | 74.0 | 98.0 |
|  |  | 87.0 | 73.0 |
|  |  | 110.0 | 162.0 |
|  |  | 81.0 | 145.0 |
|  |  | 145.0 | 135.0 |
|  |  | 147.0 | 169.0 |
|  |  | 70.0 | 102.0 |
|  |  | 105.0 | 71.0 |
| Creatinine  (mg/dL) | Total | 0.71 | 0.79 |
|  |  | 0.76 | 0.88 |
|  |  | 0.87 | 0.85 |
|  |  | 0.61 | 0.63 |
|  |  | 0.58 | 0.56 |
|  |  | 0.71 | 0.67 |
|  |  | 0.83 | 0.74 |
|  |  | 0.91 | 0.81 |
|  |  | 0.86 | 0.72 |
|  |  | 1.00 | 0.85 |
|  |  | 1.00 | 0.85 |
|  |  | 0.84 | 0.85 |
|  |  | 0.68 | 0.62 |
|  |  | 1.30 | 1.26 |
|  |  | 1.60 | 1.30 |
|  |  | 0.82 | 0.73 |
|  |  | 1.20 | 1.20 |
|  |  | 0.62 | 0.70 |
|  |  | 0.86 | 0.82 |
|  |  | 1.10 | 1.05 |
|  |  | 1.11 | 1.02 |
|  |  | 0.92 | 0.80 |
|  |  | 0.68 | 0.70 |
|  |  | 1.68 | 1.34 |
| Creatinine  (mg/dL) | ≥ 1 | 1.00 | 0.85 |
|  |  | 1.00 | 0.85 |
|  |  | 1.30 | 1.26 |
|  |  | 1.60 | 1.30 |
|  |  | 1.20 | 1.20 |
|  |  | 1.10 | 1.05 |
|  |  | 1.11 | 1.02 |
|  |  | 1.68 | 1.34 |
|  | < 1 | 0.71 | 0.79 |
|  |  | 0.76 | 0.88 |
|  |  | 0.87 | 0.85 |
|  |  | 0.61 | 0.63 |
|  |  | 0.58 | 0.56 |
|  |  | 0.71 | 0.67 |
|  |  | 0.83 | 0.74 |
|  |  | 0.91 | 0.81 |
|  |  | 0.86 | 0.72 |
|  |  | 0.84 | 0.85 |
|  |  | 0.68 | 0.62 |
|  |  | 0.82 | 0.73 |
|  |  | 0.62 | 0.7 |
|  |  | 0.86 | 0.82 |
|  |  | 0.92 | 0.8 |
|  |  | 0.68 | 0.7 |
| Plasmatic  Uric acid  (mg/dL) | Total | 4.7 | 4.1 |
|  |  | 4.0 | 4.1 |
|  |  | 8.6 | 7.7 |
|  |  | 5.9 | 5.8 |
|  |  | 6.0 | 5.0 |
|  |  | 3.7 | 2.9 |
|  |  | 7.9 | 6.2 |
|  |  | 8.5 | 10.5 |
|  |  | 4.4 | 3.1 |
|  |  | 6.4 | 5.9 |
|  |  | 6.0 | 5.9 |
|  |  | 4.7 | 5.2 |
|  |  | 4.0 | 5.3 |
|  |  | 7.5 | 6.4 |
|  |  | 8.4 | 5.8 |
|  |  | 7.3 | 6.8 |
|  |  | 5.3 | 4.7 |
|  |  | 3.9 | 3.2 |
|  |  | 7.8 | 5.8 |
|  |  | 7.0 | 6.7 |
|  |  | 9.8 | 9.7 |
|  |  | 5.3 | 4.2 |
|  |  | 3.7 | 3.2 |
|  |  | 6.9 | 5.9 |
| Plasmatic  Uric acid  (mg/dL) | ≥ 6 | 8.6 | 7.7 |
|  |  | 6.0 | 5.0 |
|  |  | 7.9 | 6.2 |
|  |  | 8.5 | 10.5 |
|  |  | 6.4 | 5.9 |
|  |  | 6.0 | 5.9 |
|  |  | 7.5 | 6.4 |
|  |  | 8.4 | 5.8 |
|  |  | 7.3 | 6.8 |
|  |  | 7.8 | 5.8 |
|  |  | 7.0 | 6.7 |
|  |  | 9.8 | 9.7 |
|  |  | 6.9 | 5.9 |
|  | < 6 | 4.7 | 4.1 |
|  |  | 4 | 4.1 |
|  |  | 5.9 | 5.8 |
|  |  | 3.7 | 2.9 |
|  |  | 4.4 | 3.1 |
|  |  | 4.7 | 5.2 |
|  |  | 4 | 5.3 |
|  |  | 5.3 | 4.7 |
|  |  | 3.9 | 3.2 |
|  |  | 5.3 | 4.2 |
|  |  | 3.7 | 3.2 |
| Interval PR  (ms) | Total | 140.0 | 160.0 |
|  |  | 140.0 | 140.0 |
|  |  | 140.0 | 160.0 |
|  |  | 140.0 | 160.0 |
|  |  | 140.0 | 160.0 |
|  |  | 140.0 | 160.0 |
|  |  | 140.0 | 180.0 |
|  |  | 180.0 | 220.0 |
|  |  | 120.0 | 140.0 |
|  |  | 140.0 | 140.0 |
|  |  | 140.0 | 180.0 |
|  |  | 180.0 | 180.0 |
|  |  | 160.0 | 160.0 |
|  |  | 140.0 | 140.0 |
|  |  | 180.0 | 220.0 |
|  |  | 180.0 | 160.0 |
|  |  | 180.0 | 160.0 |
|  |  | 180.0 | 160.0 |
|  |  | 160.0 | 160.0 |
|  |  | 180.0 | 180.0 |
|  |  | 160.0 | 180.0 |
|  |  | 140.0 | 160.0 |
|  |  | 180.0 | 180.0 |
|  |  | 140.0 | 140.0 |
| Interval PR  (ms) | ≥ 160 | 180.0 | 220.0 |
|  |  | 180.0 | 180.0 |
|  |  | 160.0 | 160.0 |
|  |  | 180.0 | 220.0 |
|  |  | 180.0 | 160.0 |
|  |  | 180.0 | 160.0 |
|  |  | 180.0 | 160.0 |
|  |  | 160.0 | 160.0 |
|  |  | 180.0 | 180.0 |
|  |  | 160.0 | 180.0 |
|  |  | 180.0 | 180.0 |
|  | < 160 | 140.0 | 160.0 |
|  |  | 140.0 | 140.0 |
|  |  | 140.0 | 160.0 |
|  |  | 140.0 | 160.0 |
|  |  | 140.0 | 160.0 |
|  |  | 140.0 | 160.0 |
|  |  | 140.0 | 180.0 |
|  |  | 120.0 | 140.0 |
|  |  | 140.0 | 140.0 |
|  |  | 140.0 | 180.0 |
|  |  | 140.0 | 140.0 |
|  |  | 140.0 | 160.0 |
|  |  | 140.0 | 140.0 |
| Interval QRS  (ms) | Total | 100.0 | 100.0 |
|  |  | 90.0 | 90.0 |
|  |  | 90.0 | 90.0 |
|  |  | 80.0 | 80.0 |
|  |  | 90.0 | 80.0 |
|  |  | 80.0 | 80.0 |
|  |  | 80.0 | 90.0 |
|  |  | 110.0 | 130.0 |
|  |  | 80.0 | 90.0 |
|  |  | 100.0 | 100.0 |
|  |  | 80.0 | 90.0 |
|  |  | 80.0 | 80.0 |
|  |  | 90.0 | 90.0 |
|  |  | 100.0 | 110.0 |
|  |  | 110.0 | 130.0 |
|  |  | 100.0 | 110.0 |
|  |  | 90.0 | 100.0 |
|  |  | 110.0 | 120.0 |
|  |  | 90.0 | 90.0 |
|  |  | 90.0 | 90.0 |
|  |  | 90.0 | 100.0 |
|  |  | 90.0 | 90.0 |
|  |  | 100.0 | 100.0 |
|  |  | 100.0 | 100.0 |
| Interval QRS  (ms) | ≥100 | 100.0 | 100.0 |
|  |  | 110.0 | 130.0 |
|  |  | 100.0 | 100.0 |
|  |  | 100.0 | 110.0 |
|  |  | 110.0 | 130.0 |
|  |  | 100.0 | 110.0 |
|  |  | 110.0 | 120.0 |
|  |  | 100.0 | 100.0 |
|  |  | 100.0 | 100.0 |
|  | <100 | 90.0 | 90.0 |
|  |  | 90.0 | 90.0 |
|  |  | 80.0 | 80.0 |
|  |  | 90.0 | 80.0 |
|  |  | 80.0 | 80.0 |
|  |  | 80.0 | 90.0 |
|  |  | 80.0 | 90.0 |
|  |  | 80.0 | 90.0 |
|  |  | 80.0 | 80.0 |
|  |  | 90.0 | 90.0 |
|  |  | 90.0 | 100.0 |
|  |  | 90.0 | 90.0 |
|  |  | 90.0 | 90.0 |
|  |  | 90.0 | 100.0 |
|  |  | 90.0 | 90.0 |
| Interval QT  (ms) | Total | 390.0 | 380.0 |
|  |  | 390.0 | 370.0 |
|  |  | 380.0 | 400.0 |
|  |  | 360.0 | 360.0 |
|  |  | 390.0 | 400.0 |
|  |  | 400.0 | 380.0 |
|  |  | 390.0 | 400.0 |
|  |  | 380.0 | 380.0 |
|  |  | 380.0 | 390.0 |
|  |  | 400.0 | 380.0 |
|  |  | 360.0 | 420.0 |
|  |  | 400.0 | 400.0 |
|  |  | 400.0 | 440.0 |
|  |  | 380.0 | 400.0 |
|  |  | 390.0 | 380.0 |
|  |  | 400.0 | 400.0 |
|  |  | 380.0 | 400.0 |
|  |  | 400.0 | 400.0 |
|  |  | 380.0 | 400.0 |
|  |  | 360.0 | 380.0 |
|  |  | 380.0 | 400.0 |
|  |  | 360.0 | 400.0 |
|  |  | 420.0 | 420.0 |
|  |  | 360.0 | 400.0 |
| Interval QT  (ms) | ≥ 380 | 390.0 | 380.0 |
|  |  | 390.0 | 370.0 |
|  |  | 380.0 | 400.0 |
|  |  | 390.0 | 400.0 |
|  |  | 400.0 | 380.0 |
|  |  | 390.0 | 400.0 |
|  |  | 380.0 | 380.0 |
|  |  | 380.0 | 390.0 |
|  |  | 400.0 | 380.0 |
|  |  | 400.0 | 400.0 |
|  |  | 400.0 | 440.0 |
|  |  | 380.0 | 400.0 |
|  |  | 390.0 | 380.0 |
|  |  | 400.0 | 400.0 |
|  |  | 380.0 | 400.0 |
|  |  | 400.0 | 400.0 |
|  |  | 380.0 | 400.0 |
|  |  | 380.0 | 400.0 |
|  |  | 420.0 | 420.0 |
|  | < 380 | 360.0 | 360.0 |
|  |  | 360.0 | 420.0 |
|  |  | 360.0 | 380.0 |
|  |  | 360.0 | 400.0 |
|  |  | 360.0 | 400.0 |
