# Supplementary material for: Data of Nebivolol on oxidative stress parameters in hypertensive patients
Source: Data Brief. 2022 Feb 3;41:107913. doi: 10.1016/j.dib.2022.107913 (PMC8847804; doi:10.1016/j.dib.2022.107913)
Supplement: Supplementary file 3 [file mmc3.doc]

**Supplementary Material**

| **Supplementary Table 3 – Body mass index (BMI), in kg/cm^2^, of the patients Untreated (before) and Treated (after) with Nebivolol. Raw data collected.** | | | |
| --- | --- | --- | --- |
| BMI  (Kg/cm^2^) | Total | Untreated | Treated |
|  |  | 41.7 | 42.1 |
|  |  | 22.1 | 21.4 |
|  |  | 29.9 | 29.0 |
|  |  | 25.3 | 23.6 |
|  |  | 32.0 | 30.1 |
|  |  | 21.5 | 20.7 |
|  |  | 35.8 | 33.6 |
|  |  | 37.0 | 36.6 |
|  |  | 26.1 | 26.1 |
|  |  | 35.3 | 34.6 |
|  |  | 31.6 | 31.2 |
|  |  | 28.8 | 27.6 |
|  |  | 25.2 | 25.6 |
|  |  | 36.0 | 34.2 |
|  |  | 29.3 | 27.0 |
|  |  | 35.1 | 34.5 |
|  |  | 25.5 | 23.8 |
|  |  | 32.0 | 31.2 |
|  |  | 32.0 | 30.2 |
|  |  | 28.7 | 26.7 |
|  |  | 27.8 | 27.1 |
|  |  | 34.0 | 35.2 |
|  |  | 26.9 | 24.7 |
|  |  | 24.1 | 23.3 |
|  |  |  |  |
| BMI  (Kg/cm^2^) | ≥ 25 | 41.7 | 42.1 |
|  |  | 29.9 | 29.0 |
|  |  | 25.3 | 23.6 |
|  |  | 32.0 | 30.1 |
|  |  | 35.8 | 33.6 |
|  |  | 37.0 | 36.6 |
|  |  | 26.1 | 26.1 |
|  |  | 35.3 | 34.6 |
|  |  | 31.6 | 31.2 |
|  |  | 28.8 | 27.6 |
|  |  | 25.2 | 25.6 |
|  |  | 36.0 | 34.2 |
|  |  | 29.3 | 27.0 |
|  |  | 35.1 | 34.5 |
|  |  | 25.5 | 23.8 |
|  |  | 32.0 | 31.2 |
|  |  | 32.0 | 30.2 |
|  |  | 28.7 | 26.7 |
|  |  | 27.8 | 27.1 |
|  |  | 34.0 | 35.2 |
|  | < 25 | 26.9 | 24.7 |
|  |  | 22.1 | 21.4 |
|  |  | 21.5 | 20.7 |
|  |  | 24.1 | 23.3 |
